# Herbivore Body Condition Response in Altered Environments: Mule Deer and Habitat Management

**DOI:** 10.1371/journal.pone.0106374

**Published:** 2014-09-03

**Authors:** Eric J. Bergman, Paul F. Doherty, Chad J. Bishop, Lisa L. Wolfe, Bradley A. Banulis

**Affiliations:** 1 Mammals Research, Colorado Parks and Wildlife, Fort Collins, Colorado, United States of America; 2 Department of Fish, Wildlife, and Conservation Biology, Colorado State University, Fort Collins, Colorado, United States of America; 3 Terrestrial Programs, Colorado Parks and Wildlife, Montrose, Colorado, United States of America; Auburn University, United States of America

## Abstract

The relationships between habitat, body condition, life history characteristics, and fitness components of ungulates are interwoven and of interest to researchers as they strive to understand the impacts of a changing environment. With the increased availability of portable ultrasound machines and the refinement of hormonal assays, assessment of ungulate body condition has become an accessible monitoring strategy. We employed body condition scoring, estimation of % ingesta-free body fat (%IFBF), assessment of free thyroid hormones (FT4 and FT3), and assessment of pregnancy, as metrics to determine if landscape-level habitat treatments affected body condition of adult (≥1.5 years old) female mule deer (*Odocoileus hemionus*). All body condition related metrics were measured on 2 neighboring study areas — a reference area that had received no habitat treatments and a treatment study area that had received mechanical removal of pinyon pine (*Pinyus edulis*) - Utah juniper (*Juniperus osteosperma*) forest, chemical control of weeds, and reseeding with preferred mule deer browse species. A consistent trend of higher %IFBF was observed in the treatment study area 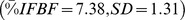 than in the reference study area 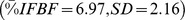, although variation of estimates was larger than hypothesized. A similar pattern was observed with higher thyroid hormones concentrations being observed in the treatment study area, but large amounts of variation within concentration estimates were also observed. The consistent pattern of higher body condition related estimates in our treatment study area provides evidence that large mammalian species are sensitive to landscape change, although variation within estimates underlie the challenge in detecting population level impacts stemming from environmental change.

## Introduction

Natural succession, climate mediated habitat change, deliberate habitat improvement, and direct habitat loss result in changing environments for wildlife populations. Due to the economic and social value of large ungulates, and in turn the datasets that management of these species foster, large ungulate populations are attractive to researchers hoping to elucidate the impacts of environmental change. Yet while wildlife professionals hasten to document the impacts of environmental change, the best barometer for measuring impacts to individuals and populations remains elusive. In general, the cascading effect of habitat quantity and quality on wildlife fitness has received attention for several decades [1]–[5]. Specifically, in bottom-up systems, the predicted sequence of density-dependent effects experienced by mammals as their populations saturate a landscape and approach the local carrying capacity have been succinctly predicted [1], [2]: 1) reduced survival of juveniles, 2) delay in age of first pregnancy, 3) reduced neonatal and parturition rates of adults, and finally 4) reduced survival of adults. These predictions have subsequently been applied to large ungulate species [3], [4]. Of these predictions, all but the first are directly related to body condition of adult females (≥1 year old). Thus, the relationships between habitat, body condition and life history characteristics are tightly interwoven [6], [7]. Despite this broad history of investigation, the body condition and fitness of ungulates has not been used as a tool for evaluating habitat management.

The need to evaluate individual-level metrics as a response to environmental change rests on the assumption that the effects of environmental change may be subtle. These subtle effects may have long-term fitness consequences that remain undetected at short time intervals when assessed with population level monitoring strategies. The increased availability of portable ultrasound machines, coupled with the development and validation of robust body condition estimation models [8]–[10] has made the assessment of ungulate body condition, and other fitness components, accessible monitoring strategies. Similarly, thyroid hormone concentrations reflect the metabolic condition of ungulates [11]–[13], providing a window for assessing an individual's ability to cope with current environmental conditions.

Total body fat and thyroid hormones can be viewed as metrics for the same general trait, overall deer health; however, they are parameters for different processes. Total body fat estimates, generated by merging ultrasonic rump fat measurements with body condition scoring [10], reflect the energetic reserve for an individual deer. Thyroid hormone concentrations reflect the ability of deer to utilize body fat reserves. Thyroxine (T4) hormone is a product of the thyroid gland and is a precursor to the triiodothyronine (T3) hormone [14], [15]. The T3 hormone plays a direct role in regulating the basal metabolic rate and thermal regulation within animals [14], [15]. Measurements of these 2 hormones typically occur in 2 forms, total hormone concentrations (T4 and T3) and free hormone concentrations (FT4 and FT3). Variation in hormone concentrations is indicative of physiological adjustment to changes in the environment.

As managers implement habitat management actions, or as they consider alternative large scale changes to habitat (e.g., habitat response to wildfire or habitat alteration due to development), they often wish to know if ungulate populations have been affected. Experimental research has demonstrated a strong connection between maternal body condition (i.e, %IFBF and hormonal concentrations), pregnancy rates, as well as neonate and juvenile survival, when food was supplemented [7]. However, the study of Bishop et al [7] was designed to explore an ecological process, not to test a practical management scenario. In an attempt to evaluate those results [7], [13] in the context of common habitat management techniques, we conducted a study that assessed late-winter body condition of adult female mule deer with respect to such management techniques. We employed body condition scoring, estimation of total body fat and assessment of thyroid hormones, as metrics to determine if landscape-level habitat manipulation affected body condition of adult (≥1.5 years old) female mule deer. We hypothesized that estimates of these late winter condition metrics for adult females on the treatment study area would be consistent with animals in better overall condition, although we also hypothesized that our estimates would be lower than the experimentally elevated estimates reported in other research because increasing browse availability to similar *ad libitum* levels used by Bishop et al. was not a realistic expectation for our habitat management techniques [7], [13].

## Materials and Methods

### Study area

**Figure 1:**
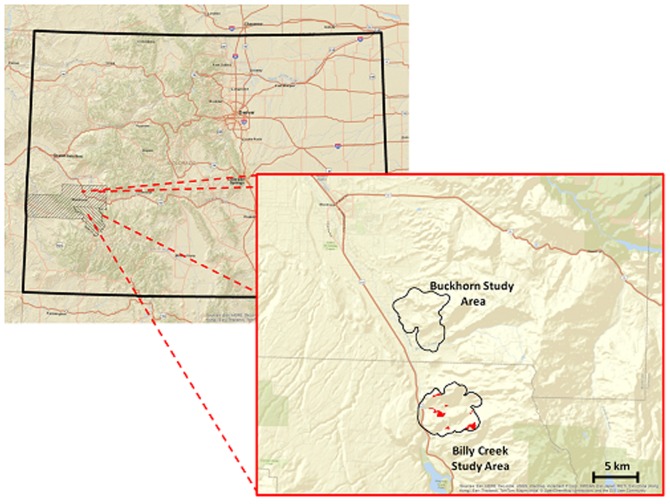
Location of 2 study areas in southwest Colorado. The Buckhorn study area was located in Montrose county, designated by hash marks from lower left to upper right. The Billy Creek study area was located in Ouray county, designated by hash marks from lower right to upper left. Habitat treatments are depicted by red polygons inside the Billy Creek study area boundaries.

We conducted this research on 2 study areas near the southeastern tip of the Uncompahgre Plateau, near the city of Montrose in southwest Colorado (38° 28.726′, 107°52.624′ – Fig. 1). One study area (Buckhorn) was maintained as a reference area, whereas the second study area (Billy Creek State Wildlife Area – BCSWA) was a treatment area (see Fig. 1). For both study areas, scientific collection was permitted by Colorado Parks and Wildlife (Licence No: CPW001 and CPW003). No component of this research involved endangered or protected species.

The study areas were located in close proximity to one another to minimize spatial variation with Buckhorn being approximately 8.5 km north of BCSWA. Each study area was located on pinyon pine - Utah juniper forest winter range. These forests were late-seral stage, typified by open understory with occasional sagebrush (*Artemisia spp.*), cliffrose (*Purshia Mexicana*), antelope bitterbrush (*Purshia tridentate*), mountain mahogany (*Cercocarpus spp.*), or rabbitbrush (*Ericameria spp.*) plants. Grasses included western wheatgrass (*Pascopyrum smithii*), green needlegrass (*Nassella viridula*), Indian ricegrass (*Achnatherum hymenoides*) and bluegrass (*Poa spp*.).

Both study areas were located within Colorado Parks and Wildlife (CPW) Data Analysis Unit (DAU) 40. This 2,437 km^2^ DAU was managed for a post-hunt population size of 13,500–15,000 mule deer. Each of these study areas was centered on public lands, although Buckhorn had private land at lower elevations. Likewise, both study areas declined in elevation from east to west. Mule deer arrival on each study area each winter was believed to have been heavily influenced by the building snowpack at higher elevations. Grazing pressure from domestic livestock was minimal on both study areas, with the majority of grazing occurring as livestock producers moved animals from summer range pastures to private pastures in the valley.

Due to the proximity of the study areas, and to the overall topography, a high degree of spatial overlap on summer range occurred among deer that used these 2 distinct winter range segments (E. Bergman, Colorado Parks and Wildlife, unpublished data). Due to this mixing on summer range, we assumed that body condition was equalized among deer prior to their arrival on our winter range study areas.

### Habitat treatments

For our research, habitat treatments occurred on BCSWA in 2 stages. The first stage occurred in 1998, during which 135.98 ha (∼5%) of the 2,688 ha study area was exposed to mechanical roller-chopper treatments (see Fig. 1). Roller-chopper treatments consisted of a large drum, affixed with perpendicular blades, that was pulled behind a bulldozer [16]. Standing trees and taller vegetation were uprooted by the bulldozer and subsequently broken into smaller pieces by the drum. On BCSWA, roller-chopper treatments ranged in size between 6.8–24.7 ha with the objective of opening the forest canopy and increasing the edge/area ratio (see Fig. 1). Treatments also created a mulched ground cover that was beneficial for holding moisture. Our second stage of habitat treatment efforts were reseeding and weed control that occurred concurrently with our study (2006–2008). Reseeding efforts targeted desirable browse species for mule deer by planting bitterbrush, cliffrose, sagebrush, serviceberry (*Amelanchier alnifolia*), and four-wing saltbush (*Atriplex canescens*). Weed control, via herbicide application, targeted cheatgrass (*Bromus tectorum*) and jointed goatgrass (*Aegilops cylindrical*). The second stage of habitat treatments occurred on 78.34 ha (∼57%) of the original treatments. The delay between the first stage of habitat treatments and the initiation of mule deer body condition monitoring was a deliberate decision to accommodate temporal variation in vegetation response, post-treatment [17]–[20]. By allowing a time lag we afforded browse species ample opportunity to grow.

### Mule deer capture and handling

During early March of each winter (2007–2009), 30 adult female deer were captured via helicopter net-gunning [21], [22]. Upon capture, all deer were immediately blind-folded, hobbled and ferried to a central processing site (≤3.2 km). At the field processing site, deer were weighed, age was estimated via tooth eruption and wear patterns [23]–[25], hind foot length was measured, and blood was drawn via jugular venipuncture. We measured the maximum subcutaneous fat thickness (cm) on the rump and the thickness of the longissimus dorsi muscle (cm) using a Sonovet 2000 (Universal Medical Systems, Bedford Hills, New York, USA) portable ultrasound machine and a 5-MHz linear transducer [8], [9], [26], [27]. We also determined a body condition score for each animal by palpating the rump [9], [10], [28]. Capture, handling and radio-collaring procedures for all aspects of this study were approved by the Institutional Animal Care and Use Committees at CPW (protocol #10-2005) and Colorado State University (protocol #08-2006A).

Body condition scores were combined with ultrasound measurements to generate a scaled estimate of the total percent of the body that was ingesta-free body fat (%IFBF) for each animal [10]. At the time of capture, pregnancy was determined via transabdominal ultrasonography [29]–[31] or via pregnancy-specific protein B concentrations [32] from blood serum samples (Biotracking, LLC, Moscow, Idaho, USA). Blood serum samples were also submitted to the Diagnostic Center for Population and Animal Health at Michigan State University (East Lansing, Michigan, USA) for estimation of T4, FT4, T3, and FT3 concentrations.

### Analytical methods

Prior to building body condition models, we tested for correlation between response variables. Based on the results of correlation analyses, we modeled 3 of the 5 body condition measurements (%IFBF, FT4, and FT3) as a response to group covariates (study area and year) and to individual covariates (chest girth, hind foot length, pregnancy status, and age). For all analyses, model selection and evaluation was based on AIC*_c_* [33]. Conditional model averaging of estimates was conducted such that average parameter estimates were generated using all models. For models in which individual parameters did not appear, β and standard error values of 0 were used. All possible combinations of additive multiple linear regression models were evaluated using the “MuMin”, and “Stats” packages in R (R Foundation for Statistical Computing, version 3.1.0. www.r-project.org, accessed 23 May 2014). While individual mass was collected for animals at the time of capture, these data were not directly used in the estimation process for %IFBF. For each of the 3 response variables, a total of 64 models were compared. These 64 models comprised a balanced model set in which all response variables were included in an equal number of models. Models containing multiplicative interactions were not included in these model sets. This modeling approach facilitated the computation and comparison of cumulative model weights for each predictor variable and response variable combination [34], [35]. To assess the effect of habitat treatments, year, %IFBF, and age on pregnancy, we modeled the probability of an individual's pregnancy status using logistic regression in the “Stats” package in R. To determine if there was evidence for a delay in age of first pregnancy, or senescence in pregnancy, second and third order polynomial models were also built. Finally, we conducted post hoc exploratory analyses to evaluate the conclusions and recommendations drawn by similar research [13] that regarded the utility of using blood serum thyroid concentrations to estimate %IFBF. This research [13] reported that the T4 and FT4 hormones were effective at predicting %IFBF (%IFBF = −4.8015–0.0946×T4+0.000603×T4^2^+0.1474×FT4+0.1426×chestgirth, R^2^ = 0.609). Following those methods [13], second and third order polynomials were allowed to occur in our later models. For all model sets, model fit was examined through residual and QQ plots. Assumptions of linear regression were upheld in all cases.

## Results

Estimated %IFBF was more correlated with T4 (0.25) and FT4 (0.18) than with T3 (0.07) and FT3 (0.09). However, the highest overall correlations were observed within categories of thyroid hormones. T4 and FT4 had the highest correlation (0.89), whereas the correlation between T3 and FT3 was slightly lower (0.70). Correlation of concentrations between the 2 T4 hormones and the 2 T3 hormones were consistently between 0.40–0.45. Correlations among predictor variables were low with the highest observed correlation occurring between individual chest girth and individual hind foot length (0.31).

The pooled, mean estimate of %IFBF for deer during this study was 7.17% (SD = 1.79). The observed mean value for BCSWA (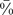 = 7.38, SD = 1.31) was higher than Buckhorn (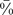 = 6.97, SD = 2.16). Overall, the effect of year was an important component of model structures for all hormones (Table 1). When %IFBF was compared among years, the mean observed estimate in 2007 (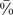 = 6.85, SD = 1.99) was less than 2008 (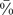 = 7.48, SD = 1.78) or 2009 (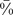 = 7.19, SD = 1.56). The observed pattern of higher %IFBF and T4 in BCSWA was observed during all 3 years, although no pattern for T3 hormone levels was observed (Table 2).

**Table 1:** Akaike's Information Criterion cumulative model weights (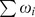), multiple linear regression coefficients 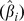, and standard errors for body condition predictor variables for adult female mule deer.

Predictor		Response Variables		
Variable		%IFBF	FT4	FT3
Unit	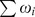	0.722	1.000	0.265
	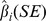	−0.385 (0.191)	−3.433 (0.614)	0.010 (0.028)
Year	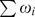	0.823	1.000	0.999
2008	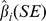	0.745 (0.273)	−3.447 (0.756)	−0.108 (0.130)
2009	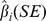	0.267 (0.267)	−5.115 (0.743)	−0.872 (0.128)
Chest	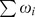	0.966	0.412	0.317
	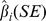	0.089 (0.028)	0.035 (0.028)	0.003 (0.003)
Age	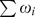	0.363	0.890	0.982
	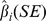	−0.025 (0.024)	−0.351 (0.138)	−0.082 (0.026)
Foot	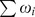	0.293	0.533	0.504
	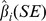	0.017 (0.027)	−0.170 (0.109)	−0.026 (0.018)
Pregnant	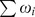	0.260	0.827	0.452
	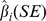	−0.016 (0.103)	−1.739 (0.754)	−0.094 (0.071)

Data were collected in southwest Colorado during early March, 2007–2009. A balanced model set was used such that all response variables appeared in an equal number of models, thus cumulative model weights >0.5 are attributed to variables that are most important.

**Table 2:** Observed mean estimates (with standard deviation) for 5 body condition variables from adult female mule deer in southwestern Colorado.

Year	Unit	%IFBF	T4	FT4	T3	FT3
2007	BCSWA	6.82 (1.51)	88.23 (15.53)	14.8 (3.98)	1.55 (0.53)	2.1 (0.7)
	Buckhorn Mountain	6.81 (2.36)	78.07 (22.34)	13.1 (4.66)	1.42 (0.31)	2.07 (0.56)
2008	BCSWA	7.91 (1.24)	94.3 (20.71)	13.37 (4.59)	1.17 (0.28)	1.98 (0.59)
	Buckhorn Mountain	7.05 (2.12)	56.17 (23.32)	8.37 (3.91)	1.17 (0.58)	2.13 (1.16)
2009	BCSWA	7.4 (0.94)	74.63 (14.61)	11.33 (3.46)	1.22 (0.32)	1.41 (0.52)
	Buckhorn Mountain	6.98 (1.99)	54.77 (19.34)	6.83 (3.17)	1.26 (0.35)	1.14 (0.44)

Data were collected during early March in a treatment study area (Billy Creek State Wildlife Area – BCSWA) and a reference study area (Buckhorn Mountain). Variables include scaled percent ingesta-free body fat (%IFBF), as well as concentrations for the thyroid hormones: T4 (nanomole/L), T3 (nanomole/L), FT4 (picomole/L), and FT3 (picomole/L).

**Figure 2:**
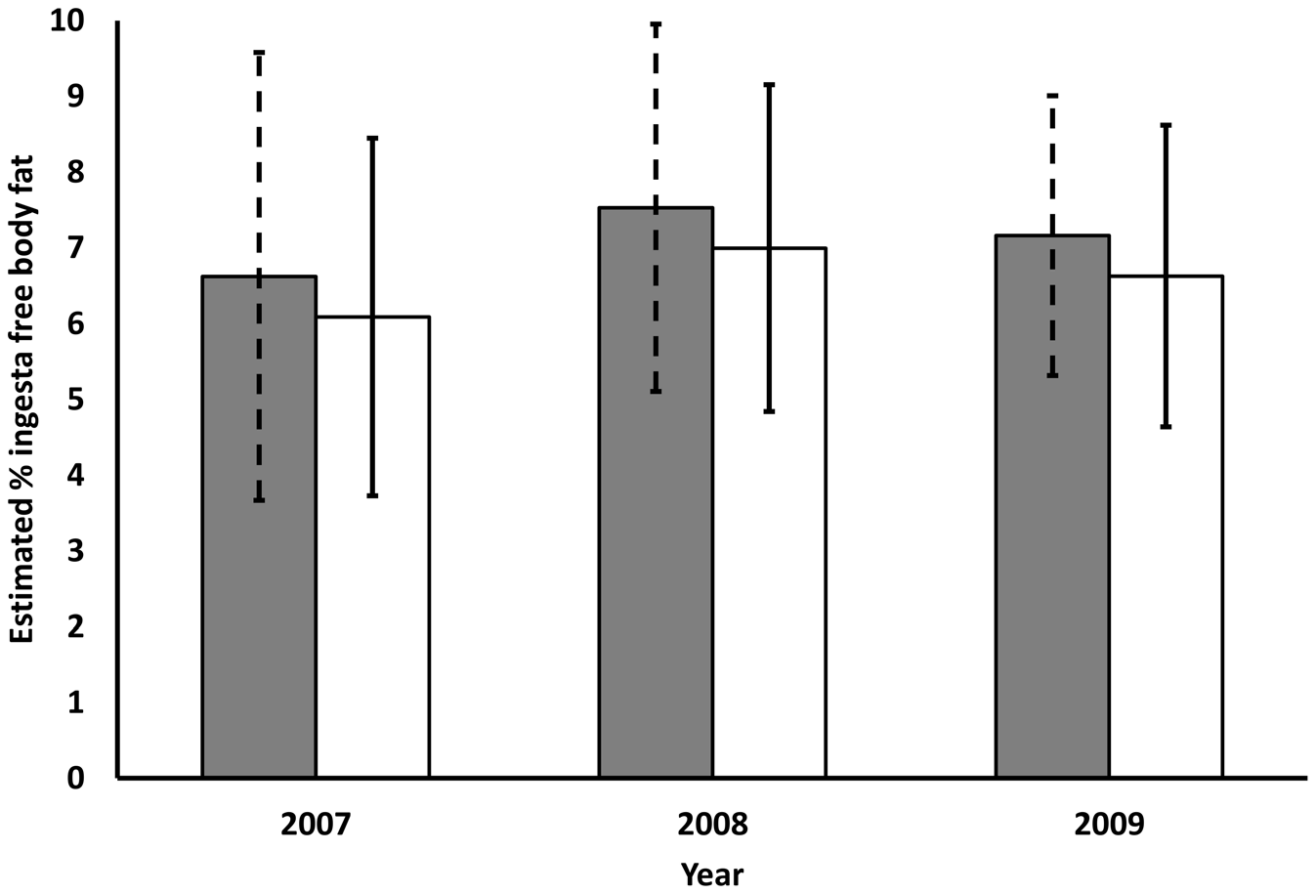
Estimates of winter body fat on treated and un-treated study areas. Scaled estimates of late winter percent ingest free body fat (%IFBF), with 95% prediction intervals, for adult female mule deer in southwest Colorado. Solid gray bars reflect estimates for our treatment study area (Billy Creek State Wildlife Area) and white bars reflect estimates for our reference study area (Buckhorn Mountain). Estimates and prediction intervals were generated according to the model 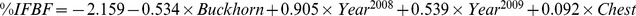 in which chest girth was held constant at the observed mean of 95.476 cm and coefficient estimates have been model averaged based on model results.

The difference in %IFBF between study areas and among years was subtle, with wide overlap in the estimates of variance (Fig. 2). The overall best model incorporated study area, year and individual chest girth (Table 3) and cumulative AIC*_c_* weights from %IFBF models also reflected the importance of these 3 covariates (see Table 1). Estimated %IFBF was higher in BCSWA than in Buckhorn, and reflected a 1.08× magnitude increase when pooled over 3 years. However, the performance of our best %IFBF model was not strong (see Table 3). Much of the variation within these data (>90%) remained unexplained. Based on cumulative AIC*_c_* weights, the remaining covariates of interest (pregnancy status, hind foot length and age) accounted for less than 0.50 and contributed little to overall model predictions (see Table 1).

**Table 3:** Best predictive multiple linear regression models, based on Akaike's Information Criteria (AIC*_c_*), for 3 different body condition related parameters in adult female mule deer in southwest Colorado.

Model	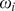 a	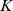 b	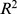
%IFBF = Area + Year + Chest	0.222	6	0.096
FT4 = Area + Year + PRG + Age	0.233	7	0.360
FT3 = Year + Age	0.141	5	0.269

a AIC*_c_* model weight.

b Number of estimated parameters.

Body condition parameters included % ingesta-free body fat (%IFBF), concentration of FT4 thyroid hormones (FT4), and concentrations of FT3 thyroid hormones (FT3). Data were modeled using study area (Area), year (Year), individual pregnancy status (PRG), chest girth (Chest), age (Age) and hind foot length (Foot).

Our linear regression models for FT4 were similar to those of %IFBF. The best FT4 model was composed of study area, year, age, and pregnancy status (see Table 3), and estimated concentrations of FT4 for Buckhorn were consistently lower than those estimated for BCSWA (see Table 1 and Table 2). However, while the highest %IFBF was estimated during the second year of the study (2008), FT4 concentrations were highest during the first year of the study. The model-averaged parameter estimate for pregnancy status in FT4 models was negative (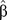 = −1.739, SE = 0.754), and a common factor in many of our top models (see Table 1). The morphometric measurements of chest girth and hind foot length explained little of the variation in our data and accounted for cumulative AIC*_c_* weights were near 0.50 (see Table 1).

The best linear regression model for FT3 deviated from the patterns established by %IFBF and FT4. The role of year and age had the greatest influence on model predictions for FT3, whereas study area did not (see Table 1). Concentration of FT3 was lower during 2008 and 2009, following the pattern observed for FT4.

**Figure 3:**
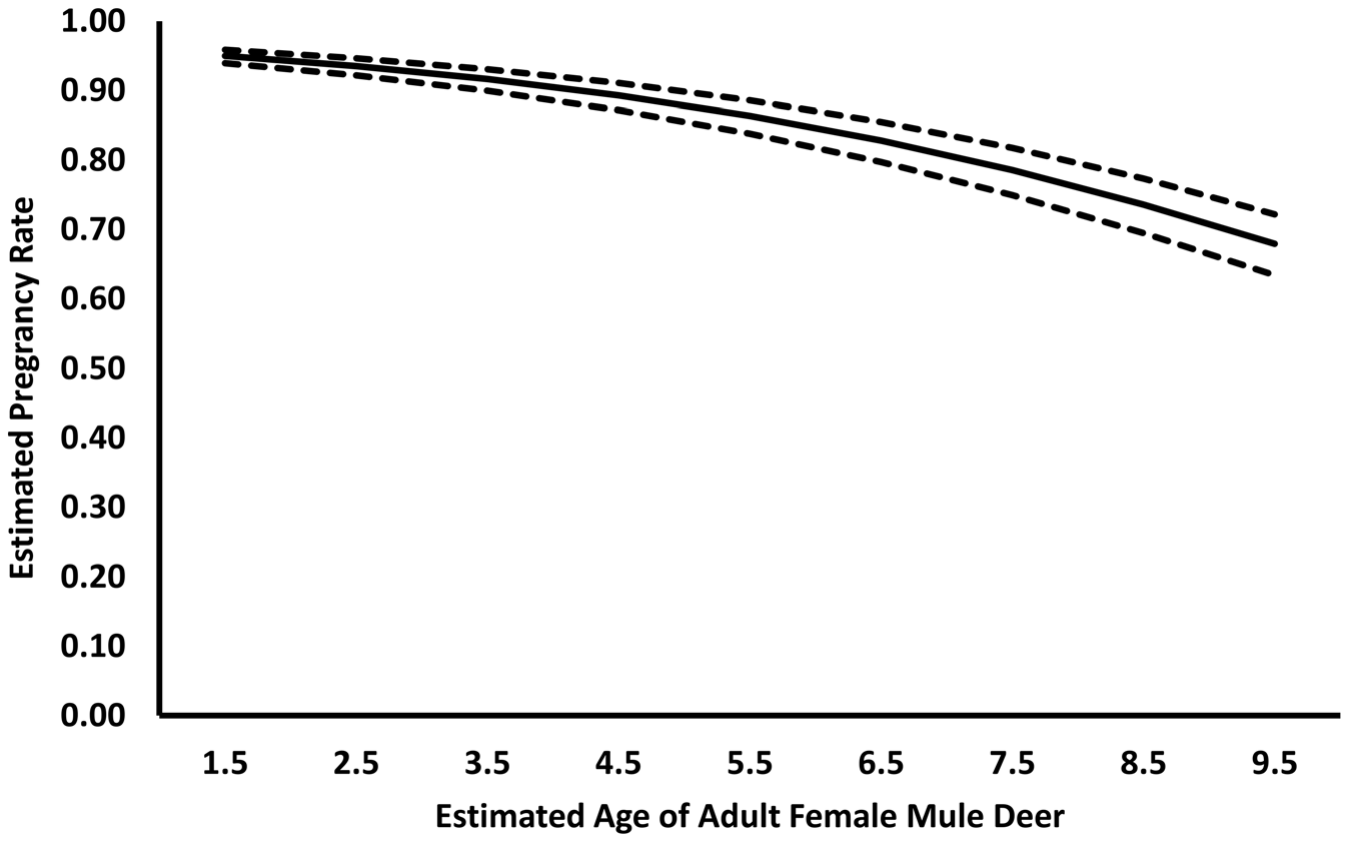
Probability of pregnancy for different age classes of adult female mule deer, with 95% confidence intervals, for adult mule deer in southwest Colorado. No discernible difference in probability of pregnancy between our treatment and reference study areas was observed, although evidence for senescence in older age classes was observed.

Based on AIC*_c_*, as well as model-averaged parameter estimates, when pregnancy status was treated as a dependent variable there was no evidence that the probability of an adult female deer being pregnant varied between study areas or during years. Little difference in pregnancy rates was observed between BCSWA and Buckhorn during the 3 year period (BCSWA = 0.877 (SD = 0.329), Buckhorn  = 0.862 (SD = 0.345)). When pooled across study areas, observed mean pregnancy was lower in 2008 than in 2007 and 2009 (2007: 0.896 (SD = 0.307), 2008: 0.833 (SD = 0.376), 2009: 0.883 (SD = 0.324)). Probability of being pregnant was best predicted by the model 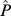 = 3.354–0.2739×age, with the effect of age on pregnancy being negative (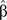 = −0.311, SE = 0.109). Our data did reflect some evidence for late age senescence (Fig. 3). Exploratory models that were structured with second and third order polynomial expressions in an attempt to accommodate delayed age of first pregnancy or late age senescence did not improve upon the simpler additive models.

Results of our exploratory analysis in which %IFBF was modeled using thyroid hormones were not congruent with results from earlier research [7]. For our data, %IFBF was best predicted by the model 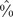
IFBF = 1.911+0.1814×TT4−0.002×TT4^2^+0.000007×TT4^3^. However, the predictive ability of our model was quite low (R^2^ = 0.106). When the model from similar research [7] was fit to our data 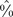
IFBF = −1.359+0.075×TT4−0.003×TT4^2^−0.050×FT4+0.058×chestgirth, R^2^ = 0.120), the model only received 2.6% of the model weight and had low predictive ability.

## Discussion

The patterns reflected by our data tend to support our hypothesis that late winter body condition of adult female mule deer was elevated in our treatment study area as compared to our reference area. Both total fat reserves (%IFBF) and the metabolic capacity to utilize those reserves (FT4) appeared to be higher in treatment deer than in reference deer. However, the considerable variation that occurred within those estimates tempers this conclusion.

For both %IFBF and FT4 results, study area and year were consistently among the most important covariates. In the case of FT4, these covariates accounted for >99% of the cumulative model weight (see Table 1), demonstrating that these covariates were most useful in explaining variation in the data. In the case of %IFBF, the single covariate that explained most of the variation in the data was chest girth. Chest girth, a variable directly related to body size, helped distinguish between large bodied animals that had low %IFBF and small bodied animals that had high %IFBF. Annual variability in body condition among winters was expected to be an important factor in assessing late winter body condition, although its importance relative to habitat management efforts was difficult to predict prior to our study. This expectation was met as yearly variation appeared in most of the best models and never carried less than 82% of the cumulative AIC*_c_* weight.

We suspect that had we been able to increase the positive effects of habitat treatments, the importance of yearly variation may have been diminished. However, the treatments delivered as part of our research reflect those commonly utilized by land management agencies. We also note that >90% of the variation within our %IFBF data remained unexplained. Much of this variation was likely due to individual characteristics (i.e., past reproductive success or failure, energetic burdens due to lactation, and habitat selection behaviors at micro-scales). Our study did not evaluate these important sources of variation. Thus, we conclude that while the effect of habitat management techniques are positive, thereby elevating the late winter body condition of mule deer, the magnitude of those effects are subtle and not be strong enough to eliminate the roles of yearly or individual variation. While the variation surrounding our estimates limited our ability to make a robust conclusion, this variation also serves as a road map for future research. Specifically, exploration of the components of this variation is warranted. Spatial and temporal aspects of this variation, largely regulated by annual moisture and weather patterns, need to be better understood. We echo the sentiments of others [5], [36] that long-term, large-scale, individual-based studies are needed. We also recognize that our research evaluated a single set of treatments (i.e., no spatial replication) with limited temporal replication, thereby introducing the potential for confounding factors. By spatially pairing our study units we attempted to control for issues associated with environmental stochasticity (i.e., extreme weather events, geological and vegetational attributes associated at micro-scales, migratory behavioral characteristics displayed by deer within a single herd). Despite these efforts, these confounding factors likely influenced our results. Similarly, we believe the wide variation in our data would have been better explained by past reproductive history. The burden, or lack thereof, stemming from lactation and energetic transfer from a dam to 1–2 offspring could not be estimated as part of this study.

Despite design limitation, one of our key objectives was to evaluate the utility of body condition metrics in the context of actual habitat treatments, as opposed to artificial feeding utilized by other studies [7]. In general, the mechanical habitat treatments utilized as part of our research did mirror the pattern stemming from the pelleted food ration [7], but as expected, the magnitude of our treatment effect was lower and more tenuous when variation in estimates was considered. The research of Bishop et al. [7] reported %IFBF estimates of 10.21%–13.90% in treatment units and 6.64%–7.60% in control units, reflecting a ∼1.61× magnitude increase. We detected a 1.08× magnitude increase using common habitat management techniques. Our results do not support the recommendations of Bishop et al. [13] that thyroid hormones could be used to estimate %IFBF as even our best predictive model did not attain a satisfactory level of performance and our overall correlation between %IFBF and hormones were low. The fact that our results do not fully validate these earlier results [13] is noteworthy as it advances our knowledge about the power and utility of body condition as a metric for assessing the impacts of environmental change.

Population-level impacts stemming from the differences in body condition on our study areas were likely nominal. For example, we did not detect a meaningful difference in pregnancy rates between study areas. Likewise, while our study did not assess neonatal rates we do not think the number of fetuses produced per adult female in the treated area was greater than that in the reference area. However, we note that we did not actively seek low quality habitat to serve as our reference area. Rather, the reference area was defined by pinyon-juniper winter range that had not received vegetation treatments. This allowed us to test the hypothesis that habitat manipulation and improvement could be used to improve winter range in terms of late winter body condition. The magnitude of improvement in body condition could be expected to be amplified, relative to pre-treatment levels, if habitat treatments were targeted to poor quality habitat.

In parallel research [37], the overwinter survival of mule deer fawns was measured on these and multiple other study areas during the same time period as our study. This research [37] documented a 1.15× increase in fawn survival on treated study areas, including BCSWA. When considered in tandem with fawn survival estimates [37], our results can be used to evaluate the sequence of density dependent effects experienced by mammals as their populations approach carrying capacity [1], [2]. Predictions state that survival of young is the first population parameter to reflect a response under habitat limited scenarios [1], [2]. This prediction is supported by research paralleling our study [37]. The second prediction is a delay in the onset of first pregnancy [1], [2]. While evidence for senescence in older age classes was observed, we did not detect a delay in age of first pregnancy. Despite a truncated evaluation, based on our data the sequence of density-dependent effects hypothesized by earlier research [1], [2] were likely correct. Thus, while our study provides a small step in linking environmental change with the fitness components of ungulates, it also exemplifies the need for further evaluation of the variation that is inherent within fitness components.

## Supporting Information

Appendix S1 **Data used in our analyses are available in Appendix S1.**
(PDF)
