# Supplementary material for: Herbivore Body Condition Response in Altered Environments: Mule Deer and Habitat Management
Source: PLoS One. 2014 Sep 3;9(9):e106374. doi: 10.1371/journal.pone.0106374 (PMC4153590; doi:10.1371/journal.pone.0106374)
Supplement: Appendix S1 — Data used in our analyses are available in Appendix S1. (PDF) [file pone.0106374.s001.pdf]

ID	CaptureYear	Unit	Age	Foot	Chest	Mass	scaledIFBF	Pregnant	TT4	TT3	FT4	FT3
1	2007	BC	7.5	50	101	70	7.193	Yes	111	1.5	15	1.9
2	2007	BC	4.5	48	113	79	7.585	No	94	1.5	16	2.8
3	2007	BC	9.5	45	102	60	2.574	Yes	49	0.8	9	1.7
4	2007	BC	3.5	49	95	60	4.918	Yes	108	2.8	16	3.6
5	2007	BC	4.5	47	93	54	4.528	Yes	43	0.9	8	1.6
6	2007	BC	3.5	48	95	63	7.574	Yes	109	1.6	18	2
7	2007	BC	9.5	46	93	62	8.044	No	86	1.6	15	2.3
8	2007	BC	4.5	46	94	65	6.091	Yes	79	1.5	12	2.3
9	2007	BC	4.5	49	95	64	8.465	Yes	113	2.7	17	3
10	2007	BC	3.5	46	88	51	4.137	Yes	77	1.1	11	1.2
11	2007	BC	2.5	46	88	56	5.309	Yes	65	1.2	10	1.5
12	2007	BC	6.5	44	93	59	6.091	No	83	1.6	17	2
13	2007	BC	2.5	48	96	65	5.609	Yes	98	2.1	13	2.6
14	2007	BC	5.5	45	88	53	6.373	Yes	117	2.5	20	3.7
15	2007	BC	3.5	49	91	62	8.524	Yes	124	2.2	26	3.2
16	2007	BC	9.5	46	92	60	8.044	Yes	89	1.6	16	2.7
17	2007	BC	3.5	49	96	69	8.826	Yes	80	1.4	10	1.6
18	2007	BC	7.5	47	85	57	7.613	Yes	74	1	16	1.5
19	2007	BC	5.5	46	95	64	6.091	Yes	78	1.3	15	1.8
20	2007	BC	4.5	48	97	69	7.768	Yes	103	1.3	17	1.8
21	2007	BC	3.5	48	106	69	8.329	Yes	78	1.2	14	1.9
22	2007	BC	4.5	47	103	75	6.091	Yes	91	1.7	14	1.8
23	2007	BC	3.5	46	90	57	6.636	Yes	87	2.1	13	3.2
24	2007	BC	7.5	47	93	63	5.895	Yes	87	1.3	14	1.9
25	2007	BC	3.5	47	94	70	8.826	Yes	108	1.4	21	1.5
26	2007	BC	5.5	47	89	57	7.654	Yes	79	1.3	14	1.7
27	2007	BC	3.5	47	94	69	7.768	Yes	96	1.6	15	1.9
28	2007	BC	4.5	48	95	64	7.959	Yes	67	0.7	12	1
29	2007	BC	6.5	46	95	68	7.223	Yes	107	1.9	21	1.9
30	2007	BC	4.5	50	94	70	6.872	Yes	67	1	9	1.3
31	2007	BM	2.5	47	91	55	5.895	Yes	104	1.2	19	1.6
32	2007	BM	9.5	46	97	73	8.826	Yes	65	1.1	13	1.3
33	2007	BM	3.5	48	98	61	6.872	No	111	1.4	18	2.4
34	2007	BM	6.5	50	104	76	6.727	Yes	86	1.5	10	1.7
35	2007	BM	5.5	47	100	68	8.328	Yes	100	1.6	15	2
36	2007	BM	4.5	48	108	64	14.688	Yes	65	1.8	9	2.3
37	2007	BM	6.5	49	93	65	4.137	Yes	38	1	6	1.5
38	2007	BM	6.5	45	96	59	5.841	Yes	73	1.6	11	1.7
39	2007	BM	4.5	47	101	65	6.39	Yes	42	1.7	7	2.4
40	2007	BM	4.5	46	100	64	8.826	Yes	92	1.5	19	2.5
41	2007	BM	3.5	45	97	56	6.091	Yes	109	2.1	19	3.3
42	2007	BM	9.5	48	102	75	7.122	Yes	86	1.2	14	1.9
43	2007	BM	4.5	50	101	69	6.091	Yes	91	1.8	14	2.4
44	2007	BM	4.5	47	94	63	5.895	Yes	91	1.4	14	2.3
45	2007	BM	5.5	47	95	64	5.895	Yes	75	1.6	12	2.2
46	2007	BM	6.5	47	107	69	4.137	Yes	41	1	8	1.4

47	2007	BM	4.5	48	103	63	3.941	Yes	89	1.6	23	2
48	2007	BM	4.5	46	99	64	4.137	Yes	82	1.4	11	2.1
49	2007	BM	7.5	48	105	72	10.389	Yes	60	1	9	1.7
50	2007	BM	5.5	46	102	63	3.355	Yes	24	0.8	4	1.1
51	2007	BM	4.5	47	96	63	5.895	No	87	1.6	14	3.2
52	2007	BM	3.5	48	97	63	5.895	Yes	82	1.4	15	2.4
53	2007	BM	8.5	48	102	73	9.608	No	79	1.6	12	2.2
54	2007	BM	6.5	50	109	62	7.776	Yes	82	1.2	13	1.5
55	2007	BM	6.5	47	100	61	9.608	Yes	86	1.8	12	3
56	2007	BM	4.5	46	97	60	6.091	Yes	116	1.9	23	2.8
57	2007	BM	7.5	47	102	64	4.918	Yes	77	1.2	12	1.8
58	2007	BM	2.5	48	97	58	9.217	Yes	89	1.3	17	2.3
59	2008	BC	5.5	47	93.5	56	3.941	Yes	35	0.7	5	0.7
60	2008	BC	3.5	47.2	98.5	64	7.654	Yes	71	1	9	2.2
61	2008	BC	2.5	46.5	96.5	61	7.977	Yes	108	1.9	11	3.4
62	2008	BC	6.5	47.5	97.5	68	8.914	No	103	1.2	15	1.8
63	2008	BC	5.5	48.2	97.5	66	8.826	Yes	86	1.1	10	1.5
64	2008	BC	2.5	43.8	87.5	48	6.872	Yes	124	1.2	17	2.8
65	2008	BC	2.5	47	94	65	7.272	Yes	127	1.2	19	2.6
66	2008	BC	2.5	47.5	95	63	9.217	Yes	84	1.3	12	2
67	2008	BC	2.5	43	94	57	7.423	Yes	97	1.3	12	2.4
68	2008	BC	6.5	47	95	74	8.826	Yes	93	0.6	15	2
69	2008	BC	3.5	45.5	89.5	51	8.044	Yes	94	1	14	1.7
70	2008	BC	3.5	47.5	95.5	58	7.263	Yes	102	1	11	1.2
71	2008	BC	2.5	48	91.5	60	7.984	Yes	87	1.2	9	1.5
72	2008	BC	3.5	47.5	91	67	8.826	Yes	104	1	12	1.1
73	2008	BC	6.5	46.2	89.5	56	8.826	Yes	68	0.6	6	1.3
74	2008	BC	7.5	49.5	98.5	73	9.998	Yes	83	1.1	11	2.3
75	2008	BC	5.5	45	91.5	58	7.801	No	131	1.2	22	2.3
76	2008	BC	4.5	46.5	93.5	60	8.826	Yes	103	1.2	15	2.5
77	2008	BC	3.5	47.3	94.5	65	6.481	Yes	76	0.9	10	1.3
78	2008	BC	2.5	45.5	92	64	6.091	Yes	88	1.5	12	2.3
79	2008	BC	7.5	47.5	101	76	7.899	No	134	1.4	26	1.6
80	2008	BC	2.5	44.5	86	55	6.65	Yes	89	1.3	12	2.2
81	2008	BC	3.5	48.5	98.5	73	8.694	Yes	72	1.2	9	2.1
82	2008	BC	2.5	47	93.5	58	6.872	Yes	83	1.1	12	1.7
83	2008	BC	3.5	48.5	98.5	63	8.551	Yes	101	1	16	1.6
84	2008	BC	4.5	51	94	55	6.481	No	72	1	10	1.9
85	2008	BC	2.5	50.5	93.5	59	8.826	Yes	100	1.4	15	2.4
86	2008	BC	2.5	46.5	97	58	9.608	Yes	101	1.5	17	1.8
87	2008	BC	2.5	46.5	96	65	8.149	Yes	109	1.5	20	3
88	2008	BC	3.5	45.5	94	61	8.435	Yes	104	1.5	17	2.2
89	2008	BM	8.5	46	94.5	61	6.872	Yes	41	1.1	6	1.5
90	2008	BM	2.5	46	90.5	59	6.872	Yes	59	0.8	8	1.4
91	2008	BM	7.5	47	93	63	2.964	No	52	0.6	7	1
92	2008	BM	7.5	49	94	61	4.918	No	43	0.8	7	1.4
93	2008	BM	5.5	47.5	96.5	63	8.826	Yes	64	1.4	8	1.9

94	2008	BM	5.5	47.5	93.5	70	5.895	Yes	41	2.9	7	5.8
95	2008	BM	1.5	49.5	90.5	53	7.654	Yes	45	1.4	7	3
96	2008	BM	4.5	47	88	57	4.528	Yes	62	2.1	9	4.3
97	2008	BM	9.5	48	90.5	62	4.918	Yes	22	0.7	5	1
98	2008	BM	10	47	87.5	57	3.355	Yes	9	0.4	3	0.7
99	2008	BM	8.5	46.5	94	62	9.608	Yes	48	0.7	8	1.2
100	2008	BM	5.5	48.5	94.5	60	8.435	No	40	0.9	8	2
101	2008	BM	4.5	49	99	64	9.608	Yes	59	1.1	10	1.5
102	2008	BM	3.5	47.5	97	56	6.481	No	66	1	9	1.7
103	2008	BM	4.5	46	94	60	9.608	No	56	0.9	8	1.5
104	2008	BM	3.5	46	95	58	6.091	Yes	101	1.2	13	1.9
105	2008	BM	2.5	48.5	93	67	10.389	Yes	70	0.7	11	2
106	2008	BM	4.5	47.1	97.7	66	6.872	Yes	50	1.1	6	1.9
107	2008	BM	3.5	49.5	91.5	65	6.39	Yes	58	0.9	6	1.3
108	2008	BM	4.5	47.9	94.2	52	8.826	Yes	63	1	7	1.5
109	2008	BM	6.5	47	98.5	59	5.895	Yes	41	0.7	5	1.6
110	2008	BM	5.5	48.5	90.5	60	3.355	Yes	27	0.4	4	0.9
111	2008	BM	4.5	48.5	101	75	11.171	Yes	49	1.3	6	2.8
112	2008	BM	3.5	48	99.5	70	8.708	Yes	46	1.1	6	2.8
113	2008	BM	5.5	47.2	97.5	66	6.481	Yes	80	1.2	11	1.4
114	2008	BM	2.5	48	95	62	6.091	Yes	91	2	15	3.6
115	2008	BM	3.5	47	97	63	8.494	Yes	125	2	23	3.6
116	2008	BM	2.5	46	89.5	57	6.481	Yes	66	1.5	8	2
117	2008	BM	9.5	46	92	51	6.872	No	77	1.4	14	3.7
118	2008	BM	3.5	44.2	92.5	56	8.826	Yes	34	1.9	6	3
119	2009	BC	2.5	49.5	106	78	7.083	No	81	0.4	11	1.6
120	2009	BC	1.5	47	89	51	6.872	No	97	1.3	14	1.1
121	2009	BC	7.5	49.5	94	57	7.423	Yes	64	1.5	9	1.5
122	2009	BC	2.5	48	90.8	55	8.826	Yes	63	1.6	8	1.5
123	2009	BC	3.5	49	102.5	72	7.263	Yes	68	1.3	9	1.7
124	2009	BC	5.5	48	103	73	7.69	Yes	85	1.1	22	1.6
125	2009	BC	3.5	47	103	60	7.788	Yes	76	1	15	1.4
126	2009	BC	2.5	46	86.5	55	6.872	Yes	55	0.8	8	0.9
127	2009	BC	3.5	47	97	64	8.252	Yes	65	1.4	9	1.6
128	2009	BC	7.5	48	96	62	8.826	Yes	103	1.4	17	1.7
129	2009	BC	4.5	48.5	97.5	65	5.895	Yes	92	1.2	14	1.9
130	2009	BC	3.5	46	94	64	6.872	Yes	63	1.1	9	1
131	2009	BC	2.5	45.5	91	52	8.044	Yes	98	2.1	13	3.2
132	2009	BC	8.5	49.5	98.5	62	6.091	Yes	49	0.7	7	0.9
133	2009	BC	5.5	47	96	64	7.849	Yes	71	1.2	9	1.3
134	2009	BC	3.5	47.5	91	59	7.263	Yes	100	1.7	17	2
135	2009	BC	5.5	46	91	54	7.635	Yes	76	0.8	12	0.9
136	2009	BC	4.5	46	90.5	60	6.481	Yes	54	1	7	1
137	2009	BC	3.5	47	93.5	56	5.895	Yes	77	1.2	12	1.2
138	2009	BC	5.5	49	92.5	72	7.849	Yes	62	1.1	8	1.1
139	2009	BC	5.5	47.5	95.5	61	8.826	No	67	1.1	11	1
140	2009	BC	4.5	47.5	96.5	63	6.793	No	55	1.4	8	2.2

141	2009	BC	5.5	46	90	53	8.826	Yes	70	1	10	0.6
142	2009	BC	1.5	44	91.5	45	7.263	Yes	83	1.3	12	0.9
143	2009	BC	3.5	49	94	59	8.576	Yes	65	1.2	10	1.1
144	2009	BC	4.5	47	97.5	60	6.872	Yes	91	1.2	11	1.9
145	2009	BC	2.5	47.75	95	62	6.091	Yes	85	1.5	16	1.9
146	2009	BC	3.5	49	94.5	64	8.35	Yes	68	1.1	10	1.3
147	2009	BC	4.5	45	98	64	7.849	Yes	76	1.3	11	1.2
148	2009	BC	2.5	47	96	63	5.7	Yes	80	1.5	11	1
149	2009	BM	3.5	48	98	62	7.263	Yes	55	1.4	8	1.3
150	2009	BM	7.5	46	94.5	60	5.895	Yes	42	1.1	5	0.7
151	2009	BM	8.5	46	92	53	3.355	Yes	52	2	6	0.9
152	2009	BM	6.5	47.5	105	69	7.932	Yes	42	1.4	4	1.5
153	2009	BM	3.5	45	90.5	54	4.918	Yes	62	1.3	8	1.6
154	2009	BM	3.5	49.5	101	66	6.091	Yes	40	1	4	1.1
155	2009	BM	4.5	50	98	71	5.7	Yes	60	1	6	0.8
156	2009	BM	5.5	47.5	92	66	8.143	Yes	52	1.2	6	1.4
157	2009	BM	4.5	48	93	63	5.895	Yes	82	1	14	0.8
158	2009	BM	3.5	46	98.5	63	5.895	Yes	60	1.4	7	1.3
159	2009	BM	6.5	45	100	62	7.849	Yes	26	1.2	3	1
160	2009	BM	2.5	49.5	95.5	69	5.895	Yes	84	1.2	9	0.9
161	2009	BM	4.5	49	96.5	59	7.849	Yes	45	1.2	6	0.9
162	2009	BM	3.5	48	94	57	5.7	Yes	28	0.9	4	0.6
163	2009	BM	9.5	49	93	58	7.849	No	74	1.5	10	0.9
164	2009	BM	3.5	47.5	92	56	8.826	Yes	30	1	4	0.9
165	2009	BM	4.5	45	99.4	63	8.826	Yes	98	1.3	15	1.4
166	2009	BM	3.5	48	100	71	8.826	Yes	68	0.8	9	0.6
167	2009	BM	6.5	46	94.5	61	14.688	Yes	81	1.2	13	0.9
168	2009	BM	5.5	46	101.6	63	8.258	No	74	1.8	10	1.9
169	2009	BM	9.5	48.2	87.5	56	4.918	Yes	15	0.4	3	0.5
170	2009	BM	9.5	45	90.5	59	5.895	Yes	43	0.9	6	0.5
171	2009	BM	4.5	48	94.5	56	6.091	Yes	72	1.4	7	1.2
172	2009	BM	4.5	46	96.4	57	6.091	Yes	47	0.9	4	0.8
173	2009	BM	8.5	47.5	95	63	8.826	No	53	1.6	5	1.4
174	2009	BM	1.5	45	85	46	6.872	Yes	64	1.5	7	1.3
175	2009	BM	6.5	44	95	67	5.895	Yes	46	1.2	4	2.3
176	2009	BM	4.5	46	93	47	6.872	Yes	41	1.5	5	1.9
177	2009	BM	3.5	48	92	60	6.091	Yes	39	1.7	4	1.5
178	2009	BM	5.5	43	90.5	50	6.091	Yes	68	1.9	9	1.4
